# Electrical stimulation of the dorsal clitoral nerve in the treatment of idiopathic defecatory urgency. A pilot study

**DOI:** 10.1007/s10151-023-02752-y

**Published:** 2023-01-17

**Authors:** N. Qvist, U. D. Hansen, P. Christensen, N. M. J. Rijkhoff, N. Klarskov, J. Duelund-Jakobsen

**Affiliations:** 1 Research Unit for Surgery, Odense University Hospital Odense, Odense, Denmark; 2 University of Southern Denmark, Odense, Denmark; 3 Research Unit for Gynecology and Obstetrics, Odense University Hospital, Odense, Denmark; 4 Department of Surgery, Pelvic Floor Clinic, Aarhus University Hospital, Aarhus, Denmark; 5 Department of Gynecology and Obstetrics, Herlev Hospital, Herlev, Denmark; 6 Department of Clinical Medicine, University of Copenhagen, Copenhagen, Denmark

**Keywords:** Dorsal clitoral nerve, Electric stimulation, Fecal urgency

## Abstract

**Purpose:**

To investigate the effect of dorsal clitoral nerve stimulation (DCNS) on bothersome urgency to defecate with or without fecal incontinence and the patient-reported discomfort or adverse effect with the method.

**Methods:**

For dorsal clitoral nerve stimulation, a battery powered, handheld stimulator was used, set to a pulse width of 200 µs and a frequency of 20 Hz. One electrode was placed at the preputium of the clitoris and acted as cathode while an anode electrode was placed on the belly. Prior to stimulation the patients were asked to complete a bowel habit diary throughout 14 consecutive days before and during stimulation.

**Results:**

Fourteen out of the 16 patients included completed the study. A decrease in the number of episodes (per day) with strong urgency declined in eight patients but increased in four cases during the stimulation period. An increase in episodes with moderate or mild urgency was observed in 11 and 6 cases, respectively, and a decrease in defecation without the feeling of urgency or passive incontinence decreased in two thirds of the patients. Two patients discontinued the study prematurely, on due to worsening in symptoms and one due to pelvic pain.

**Conclusion:**

Although the results may be promising, much still must be learned about the method including mode and duration of stimulation, better electrodes and more patient friendly equipment together with the development of better questionnaires to assess the patient burden of urgency.

## Introduction

Fecal urgency with the sudden need to rush to the toilet to empty the bowel is a common and troublesome problem with a great impact on the patient’s social life. It is a well-known symptom in various diseases such as inflammatory bowel disease, chronic diarrhea, constipation, and neurological disorders. More than 60% of females diagnosed with irritable bowel syndrome reported fecal urgency to occur always or most of the time [1]. Bothersome fecal urgency is also common in healthy individuals without any obvious disease, anatomical abnormalities, and normal stool consistence, referred to as idiopathic fecal urgency [2, 3]. There is no good or effective treatment for idiopathic fecal urgency and many of the individuals may also suffer from urinary urgency with or without incontinence. Whether there is a pathogenetic coincidence is unknown. Previous vaginal delivery may play an important role, as the condition is rare in males.

Electrical stimulation to treat urinary and/or fecal incontinence have been practiced for several decades either with stimulation of the sacral nerve roots (SNS) or stimulation of the posterior tibial nerve (PTNS) as the most common method. Electric stimulation of the dorsal clitoral nerve (DCNS) has been applied in small pilot studies in individuals with fecal incontinence and showed positive effect [4–6]. The dorsal clitoral nerve is the terminal branch of the pudendal nerve with important sensory and motor control of the anorectum. The nerve is superficial and easily accessible for electric stimulation.

Bowel dysfunction is a complex mixture of several symptoms with fecal urgency as the prevailing and most bothersome symptom in some of the individuals. The results on the symptom relief in fecal urgency has not been well-documented by any of the previous methods with nerve stimulation.

The aim of our study was to investigate whether a 2 week course of a daily electric stimulation of the dorsal clitoral nerve in a home-setting would change the subjective feeling of urgency as the primary outcome. Secondary outcomes were episode with fecal incontinence and patient-reported discomfort or adverse effect with the method.

## Material and methods

A total of 16 females with idiopathic fecal urgency (without obvious abnormalities at endoscopy and conventional anorectal investigation) with or without incontinence were included at Odense University Hospital and Aarhus University Hospital. Median age was 47 (range: 24–71) years. All patients had undergone an investigational program including sigmoidoscopy or colonoscopy, gynecological examination and endoanal ultrasonography to exclude any major abnormalities. Symptom duration varied from 11 months to more than 5 years without any significant complaints of diarrhea or constipation. None of the patients had previously undergone major pelvic surgery, had not received pelvic radiotherapy and had no history of diabetes or neurological disorders.

Prior to stimulation the patients were asked to complete a bowel habit diary throughout 14 consecutive days with registration of all urgency episodes and to classify whether the urgency to defecate was experienced as strong, moderate or mild or there had been no feeling of urge prior to defecation. In addition, they were asked to register whether they experienced fecal incontinence and to register stool consistence as watery, soft or solid.

For dorsal clitoral nerve stimulation, a battery powered, handheld stimulator (Beurer EM49, Beurer, Ulm, Germany) was used, set to a pulse width of 200 µs and a frequency of 20 Hz. One electrode (Ambu Neuroline 700; Ambu A/S, Ballerup, Denmark) was placed at the preputium of the clitoris and acted as cathode while an anode (Axelgaard PALS^®^; Axelgaard Manufacturing, Fallbrook, California, USA) was placed on the belly. All patients received meticulous instruction inplacing the electrodes correctly and how to operate the stimulator. The stimulator was adjusted to give the maximal tolerated amplitude at the instruction visit, and the patients were encouraged to increase the amplitude during the stimulation period if they felt confident with that or to decrease it if unpleasant.

A stimulation of 30 min duration was applied for 14 consecutive days, and the patients were asked to register the amplitude used and to register discomfort/pain experienced during stimulation on a visual analog scale. During the stimulation period they completed a bowel diary similar to the diary completed prior to stimulation. All patients were trained in correct placement of electrodes and the use of the equipment by trained research personnel at each center.

The study was approved by the scientific ethical committee of Southern Denmark (S-20190074) and informed consent was obtained from all patients.

### Statical analysis

Wilcoxon’s matched pairs test was used to compare the results before and after stimulation. For the primary outcome the number daily episodes of the four different grades of patient-reported urgencies (strong, moderate, mild, none) during a 2 week period prior to stimulation and during the period with 2 week stimulation was calculated for each patient so that each patient served as her own control. The number of urgencies were calculated per day to compensate for dates of missing report in the diary. For the episodes of incontinence, no such correction was performed due to the low numbers. For group comparison the *X*^2^ test was used. A *P* value below 0.05 was considered as statistically significant.

## Results

Fourteen out of 16 patients completed the study. Of those that did not complete the study one (71 years old) stopped due to worsening symptoms, the other (41 years old) due to unpleasant pain in the back and pelvis during stimulation. No other adverse events were observed.

**Fig. 1:**
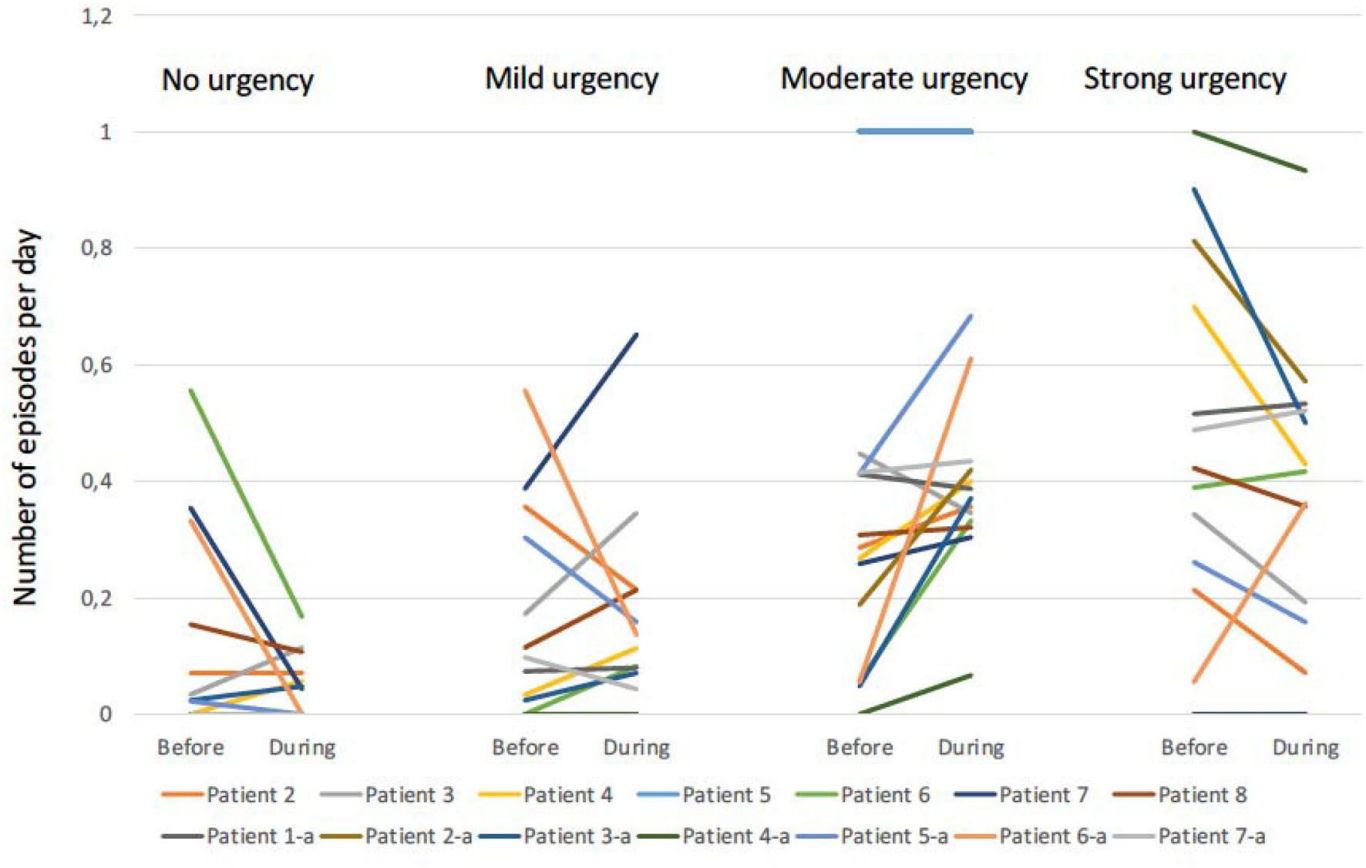
Number of reported urgencies with different strength pr day in each patient before and during dorsal clitoral nerve stimulation. Patients 2–8 from Odense Center and patients 1-a to 7-a from the Aarhus Center

A decrease in the number of episodes (per day) with strong urgency declined in 8 patients but increased in 4 during the stimulation period compared to the period before (Fig. 1). An increase in episodes of moderate or mild urgency was observed in 11 and 6 patients respectively and a decrease in defecation without the feeling of urgency or passive incontinence decreased in two thirds of the patients. This was also reflected by the overall decrease in the number of episodes of strong urgency and an increase in episodes with moderate urgency. The differences did not reach statistically significance. In three patients missing data of bowel function during stimulation was encountered varying from 1 to 3 days.

There were no significant changes in the total number of defecations with 451 episodes before and 383 during stimulation. No significant changes in stool consistency was found. Nine patients reported episodes of fecal incontinence/leakage, but only a few episodes in most of the patients. During stimulation 2 of these reported no incontinence episodes, and no significant changes was reported by 7 patients. The total number of episodes decreased from 51 to 37 during stimulation period.

The mean value of the stimulation amplitude used in each patient varied from 9.7 to 30.5 mA and with a median value of 18.6 mA. The mean value of the experienced discomfort/pain varied from 1.9 to 9.7 with a median value of 6.2 on the 10 graded VAS.

The figures were too small for a comparison of the first and second stimulation weeks. Follow-up outside the planned study period was not performed. Difficulties in keeping the clitoral electrode in place were reported by two patients. Others found the equipment/stimulator clumsy.

## Discussion

Our pilot study has shown that electrical stimulation of the clitoral nerve with the current equipment is feasible However, there are several unresolved issues. When it comes to effect there was a tendency to decrease the intensity of faecal urgency. We consider this as an relevant surrogate for a positive treatment effect. Most of the patients did not experience subjective improvement until the end of the stimulation period indicating a certain lag of time before effect.

Dorsal clitoral nerve stimulation has a documented effect on urinary urgency, prompting the current polit study to look at effect on faecal urgency. . . In order to be consistent with the other limited trials of clitoral nerve stimulation we focussed on  the effect during daily stimulation for a 2 week period  However, optimum duration and mode of stimulation remain unclear. In addition, better electrodes and more patient friendly equipment are required

Whether the effect of dorsal clitoral nerve stimuation is durable, and whether the observed effect  on  urgency placebo effect is unknown. Another concern is the number of patients experiencing pain or discomfort during the stimulation period and that two patients decided to withdraw from the study due to pain or worsening of symptoms. Patients were encouraged to set the stimulation as high as tolerated. A lower stimulation amplitude would probably have been more comfortable, but may have changed the efficacy.

Neuromodulation for fecal incontinence has been practiced for several decades. Long term results using sacral neuromodulation in patients with a wide aetiological spectrum of fecal incontinence were favorable in only half of patients and significant side effects or complications occurred in one quarter [7]. Posterior tibial nerve stimulation has been shown to decrease episodes with fecal incontinence but without any effect on the fecal incontinence score and long-term results remain unknown [8]. A major contributor to fecal incontinence is the inability to defer defecation and it is thus relevant to investigate the effect of electrical stimulation on urgency. Few studies have specifically focussed on the effect of neuromodulation on urgency.  In a study by Matzel et al. [9] the ability to postpone defecation was significantly improved after 12 months with sacral neuromodualtion. Another study showed an improvement in urgency in 47% of patients at 3 months follow-up decreasing to 20% at 48 months [10]. In the CONFIDeNT trial a significant decrease in episodes of urge fecal incontinence was observed with tibial nerve stimulation [11].

Regarding the literature on dorsal clitoral nerve stimulation, in a study by Binnie et al. [5] the clitoral-anal reflex to strengthen the anal closure mechanism was utilized. However,  this is not regular nerve modulation. A stimulus with a frequency of 1 HZ, a pulse duration of 0.1 ms with a sub-maximal tolerable stimulation voltage was used. Stimulation was applied for 5 min three times a day for eight weeks and was self-administered. A total of eight healthy females aged 32–65 yeas with 1–3 vaginal deliveries were included. All had a history of fecal incontinence impairing social life. Pre-treatment assessment included anal manometry and neurophysiological studies of pudendal-anal-reflex latency and an electromyogram of the external sphincter. Seven of the eight patients became continent for feces and with an improved maximal squeeze pressure in all and an increase in the electromyographic response of the anal sphincter. There is no long-term data. In a retrospective study including 39 females and 3 males with intractable idiopathic fecal incontinence [6] a stimulation protocol similar to Binnie et al. [5] was used. An improvement in the Wexner score from an average of 9.3 to 6 was observed. There is no idata on urgency, follow-up or how many of the patients that became completely continent. In the other study dorsal genital nerve stimulation performed twice daily for 15 min during a period of 3 weeks in nine females (two with minor sphincter defects) with fecal incontinence resulted in a significant improvement in the Wexner and St. Mark’s fecal continence score during stimulation and at 3 weeks follow-up [4]. Only one patient became fully continent. Stimulation amplitude, pulse duration and frequency were similar to the present study. In six out of nine patients the number of urgency episodes decreased and remained decreased at the 3 weeks follow-up. The Fecal Incontinence Quality of Life Score showed no significant difference. At a 1–6 months telephone follow-up all reported that their symptoms had returned to baseline levels.

Limitations of the study include the small number of patients and the observed trends in data not reaching statistical significance. To our knowledge there is no  validated questionnaire for the evaluation of the degree of urgency. Our selection of intensity of urgency is very subjective but the potential for bias is reduced by patients acting as their own controls.  An evaluation of the patient’s global experience of the treatment would have been informative but was unfortunately not a part of the study protocol. A minimum requirement for treatment effect would have been appropriate, but this would have required a much larger study.

In other  studies the duration of stimulation has been shorter but for a longer period of 3–8 weeks. The effect of different treatment programs is unknown. The low cost, the non-invasiveness and the ease of application of dorsal clitoral nerve stimulation  compared with the expensive SNS and the caregiver dependent PTNS, justify further investigation of this technique in our view. More appropriate electrodes especially for the clitoral stimulation, and more user friendly equipment for long-term stimulation have recently been developed [12].
